# How Neutrophils Shape the Immune Response: Reassessing Their Multifaceted Role in Health and Disease

**DOI:** 10.3390/ijms242417583

**Published:** 2023-12-18

**Authors:** Areez Shafqat, Jibran Ahmad Khan, Aghiad Yahya Alkachem, Homaira Sabur, Khaled Alkattan, Ahmed Yaqinuddin, Garwin Kim Sing

**Affiliations:** College of Medicine, Alfaisal University, Riyadh 11533, Saudi Arabiakkattan@alfaisal.edu (K.A.); ayaqinuddin@alfaisal.edu (A.Y.); gksing@alfaisal.edu (G.K.S.)

**Keywords:** neutrophils, adaptive immunity, neutrophil extracellular traps, antigen-presenting cells, COVID-19, tumor microenvironment, autoimmunity

## Abstract

Neutrophils are the most abundant of the circulating immune cells and are the first to be recruited to sites of inflammation. Neutrophils are a heterogeneous group of immune cells from which are derived extracellular traps (NETs), reactive oxygen species, cytokines, chemokines, immunomodulatory factors, and alarmins that regulate the recruitment and phenotypes of neutrophils, macrophages, dendritic cells, T cells, and B cells. In addition, cytokine-stimulated neutrophils can express class II major histocompatibility complex and the internal machinery necessary for successful antigen presentation to memory CD4^+^ T cells. This may be relevant in the context of vaccine memory. Neutrophils thus emerge as orchestrators of immune responses that play a key role in determining the outcome of infections, vaccine efficacy, and chronic diseases like autoimmunity and cancer. This review aims to provide a synthesis of current evidence as regards the role of these functions of neutrophils in homeostasis and disease.

## 1. Introduction

Neutrophils, or polymorphonuclear granulocytes (PMNs), are the most abundant of the circulating white blood cells and have traditionally been considered a homogenous population of cells that execute stereotypical antimicrobial effector functions. This view has been supported by the short lifespan of neutrophils and their lack of proliferative capacity. These effector functions include phagocytosis, the production of reactive oxygen species (ROS) via the respiratory burst, degranulation to release cytotoxic neutrophil granule enzymes, and release of neutrophil extracellular traps (NETs) that trap microbes to sequester them in locales of high antimicrobial activity and prevent their dissemination [1]. Through these functions, neutrophils enhance local tissue inflammation and microbial death, but, if these processes are dysregulated, exert significant collateral damage on host tissues.

This classical conception of neutrophils has largely been replaced with an understanding that considers neutrophils a heterogenous population comprised of various unique functional states capable of mounting different effector functions in health and disease. The discovery of the following functions of neutrophils has led to their consideration as orchestrators of innate and adaptive immune responses in infectious and sterile disease states. Neutrophils can produce pro- and anti-inflammatory cytokines, chemokines, and immunomodulatory factors that can modulate the recruitment and phenotypes of neutrophils, macrophages, dendritic cells (DCs), T cells, and B cells [2,3]. Although neutrophils are substantially less efficient than macrophages or DCs at producing cytokines and chemokines, their sheer abundance at sites of inflammation—several folds of magnitude greater than macrophages and DCs—suggests that they contribute significantly to shaping the inflammatory milieu and, by extension, the immune landscape at these sites. Secondly, NETs can modulate the activation of DCs, T cells, and B cells in chronic inflammatory states like autoimmune diseases and cancer [4,5]. These roles of NETs are focused on in this review instead of their well-established contribution to antimicrobial defense and thrombosis [6,7]. Lastly, cytokine-stimulated neutrophils can express class II major histocompatibility complex (MHC),costimulatory molecules, and the antigen-processing machinery required to present antigens to CD4^+^ T cells, thereby functioning as atypical APCs [8].

These functions, the continuous production of neutrophils, their abundance at sites of inflammation, and data now showing that the lifespan of neutrophils may be significantly prolonged by certain cytokines at inflammatory sites like the tumor microenvironment (TME) suggest that neutrophils, alongside macrophages and DCs, are key orchestrators of the immune response. Neutrophils are also key to wound repair after injury and a reversion to homeostasis. There is great interest in characterizing the biological implications of these aspects of neutrophil biology in not only disease but also homeostasis to reveal potential disease biomarkers and targets for therapeutic interventions.

## 2. Overview of Neutrophil Biology

### 2.1. Neutrophil Effector Functions

Neutrophils are the first immune cells recruited to sites of septic and sterile pathologies [9,10]. The three classical effector functions of neutrophils include phagocytosis, degranulation, and NET release. Phagocytosis involves engulfing cellular debris or microbial pathogens into a phagosome, which is eventually fused with granules containing microbicidal proteins [11]. NADPH oxidase embedded within the membrane of the phagosome produces reactive oxygen species (ROS) like superoxide, which is subsequently converted by azurophilic granule enzyme myeloperoxidase (MPO) into hypochlorous acid (bleach in the vernacular) that is highly bacteriotoxic [12]. The capacity of neutrophils to kill phagocytosed microbial pathogens far exceeds that of other immune cells [10]. Alternatively, neutrophils may fuse their granules with the plasma membrane to release ROS and antimicrobial granule proteins into the extracellular space [10].

Neutrophils can extrude their granule proteins within a meshwork of nuclear or mitochondrial DNA as NETs. The ‘classical’ pathway of NET production (or NETosis) starts with the activation of protein kinase C (PKC) or the RAF/MEK/ERK signaling pathways, which, in turn, activate NADPH oxidase [13,14]. The consequent cytosolic Ca^2+^ spike activates peptidylarginine deaminase-4 (PAD4), which citrullinates histone proteins to decrease electrostatic attractions between DNA and histones, leading to chromatin decondensation [15,16]. Superoxide generated by NAPDH oxidase activates MPO, which liberates neutrophil elastase (NE) from azurophilic granules [17]. MPO and NE then work synergistically to break down the nuclear envelope and cleave various cytosolic proteins. One of these proteins, gasdermin D, is a pore-forming protein that inserts into the cell membrane to cause pyroptotic cell death but additionally promotes NETosis by inserting into the plasma membrane of neutrophils [18,19]. The absolute dependency of NET formation on the various proteins discussed is debated. For example, whereas NADPH oxidase-dependent lytic NETosis takes ~3–4 h in vitro, live neutrophils can rapidly release antimicrobial NETs in an NADPH oxidase-independent manner without neutrophil cell death [20,21]. Similarly, PAD4 is indispensable for NETosis in some animal models [22], but it does not figure in immune complex (IC)-mediated NET production in autoimmune diseases like systemic lupus erythematosus (SLE) [23]. Lastly, inflammasome-independent NET formation does not require gasdermin-D activation and also proceeds without inducing cell death [24]. Interestingly, inflammasome-dependent NET formation, which activates gasdermin D, also does not necessarily induce pyroptotic cell death [24,25].

### 2.2. Metabolic Control of Neutrophil Effector Functions

Compared to other immune cells, neutrophils have a lower number of mitochondria that are also relatively smaller and have reduced activity [26,27]. Neutrophils thus rely primarily on anaerobic glycolysis for ATP production and also utilize glucose in the hexose monophosphate (HMP) shunt, which produces NADPH to fuel NADPH oxidase-dependent processes like ROS production and NETosis [28]. Hence, a large proportion of the neutrophil effector response indirectly depends on glucose metabolism [3].

Neutrophils are sensitive to changes in extracellular glucose, as evidenced by their increased propensity to undergo NADPH oxidase-dependent NETosis in diabetes [29,30,31]. Cancer is the prototypical disease where metabolic reprogramming of neutrophils can occur. Tumor cells, via the Warburg effect, augment their glucose uptake and utilization, thereby depleting extracellular glucose and increasing competition for this nutrient with stromal and immune cells residing in the TME [32,33]. Similarly, sites of autoimmune diseases are enriched in stimuli that activate effector functions of neutrophils, macrophages, T cells, and B cells, which are very metabolically demanding processes [34]. Metabolic reprogramming refers to the collective changes in immunometabolism of innate and adaptive immune cells at these sites, such as altered nutrient uptake and/or the diversion of nutrients towards certain metabolic pathways in ways that are connected to changes in immune cell effector functions [34]. Indeed, metabolic pathways are important regulators of lineage specification of immune cells (namely T cells) and their polarization towards pro-inflammatory and anti-inflammatory phenotypes (e.g., macrophages and neutrophils) [34].

Therefore, immunometabolic reprogramming of is a crucial disease-modifying factor in the biology and phenotypic outcomes of inflammatory diseases like autoimmunity, autoinflammation, and cancer. However, the multitude of factors—like cytokines, chemokines, growth factors, and metabolites—that modulate neutrophil effector functions at sites of cancer or autoimmunity makes it difficult to causally attribute their phenotypes to certain stimuli [35,36,37,38,39]. Furthermore, in vitro studies in highly controlled environments testing the effects of certain stimuli do not accurately recapitulate in vivo conditions. Nevertheless, the impacts of the metabolic reprogramming of neutrophils in the context of disease states like COVID-19, cancer, and autoimmunity are discussed in their respective sections below.

### 2.3. Neutrophil Heterogeneity

Neutrophils were traditionally considered a homogenous population of immune cells. This is no longer the case. The advent and widespread use of single-cell multi-omic approaches in the past decade has led to a paradigm shift in immunology that moves past the binary classification of immune cells like macrophages (M1 and M2) in favor of a spectrum of activation phenotypes that range between the extremes of pro- and non-inflammatory [40]. Neutrophil research lagged behind that of other immune cells mainly due to their short lifespan, their sensitivity to manipulation ex vivo, and their ability to be activated by various stimuli. However, transcriptomic studies on neutrophils under specialized protocols have revealed considerable heterogeneity at the level of bone marrow, circulation, and tissues [41,42,43,44,45,46,47].

The neutrophil is derived from the differentiation of proliferative granulocyte progenitors in the bone marrow, a process termed granulopoiesis or myelopoiesis, which takes ~2 days in mice and ~6 days in humans. Mature neutrophils are then released into the bloodstream, where their half-life is less than 24 h [48,49]. Important chemokines and associated receptors in granulopoiesis include CXCL12/CXCR4 chemoattractive interactions and VLA-4/VCAM1 adhesive interactions that retain hematopoietic progenitor cells within specific niches in the bone marrow [50,51]. G-CSF can disrupt these retentive signals to induce differentiation of granulocyte-committed precursors [50]. CXCL12/CXCR4 signaling also works to retain mature neutrophils in the bone marrow. Antagonizing this, the CXCL8/CXCR2 axis and G-CSF stimulate the release of mature neutrophils into circulation [50,51,52].

The process of neutrophil maturation in the bone marrow and release into the bloodstream features a chronological sequence of transcriptional programming and acquisition of effector microbicidal and inflammatory properties termed the neutrotime that is likely conserved in both mice and humans [53]. This produces the neutrophil heterogeneity seen during steady state/homeostatic conditions [54,55,56]. Functionally, transcriptionally distinct mature neutrophil subsets in circulation may be differentially endowed to successfully perform different effector functions, like NETosis and interferon (IFN) responses [57]. Extending this developmental timeline, neutrophils homing into different target organs like the liver, lung, spleen, and intestine—which appears to be a selective process determined by the differential expression of certain chemokine receptors or integrins in certain circulating neutrophils—are further reprogrammed in terms of their baseline and inducible gene expression signatures to acquire both organ-specific transcriptional signatures and enhanced inflammatory capacities [44,46,54]. This allows neutrophils to perform tissue-tailored tasks like adaptive immunity-related functions (e.g., antigen-presentation and B-cell help) in the spleen and vascular repair in the lungs [44,58]. During inflammation, whether or not the functions of tissue-resident neutrophils vary from infiltrating circulating neutrophils is not well understood.

Pathologies like COVID-19 and cancer or therapies like G-CSF as a treatment for neutropenia promote emergency myelopoiesis, where immature neutrophil precursors are expanded and released into the circulation to coexist with terminally differentiated PMNs [59]. This further diversifies the neutrophil population since the bone marrow population of neutrophils comprises transcriptionally distinct states capable of enacting different effector functions [53,60,61]. Immature neutrophils also undergo context-dependent transcriptomic reprogramming due to accelerated development and release from the bone marrow and according to the nature of the inciting stimulus [62]. Heterogeneity is amplified by the tremendous plasticity neutrophils display in transitioning between different transcriptional states in homeostasis and inflammation [63,64,65]. This plasticity of neutrophils indicates that the in vitro classification into rigid states like N1 and N2 [66], whose identities can be traced by expressed genes and surface markers, may not be entirely reflective of how these cells are constantly responding to and integrating environmental cues into gene expression changes. Recent studies have shown that this adaptability of inflammatory neutrophils in both mice and humans and across different tissues is controlled by a transcriptional hub of core inflammatory genes (*IL-1* family, *CD14, IL-4R, CD69*, and *PD-L1*) regulated by transcription factors like NF-κB, AP-1, and JunB [56]. Importantly, JunB depletion has been shown to subdue the expression of the core inflammatory genes in resting and activated neutrophils in the setting of myocardial infarction, thereby reducing pathological inflammation [46,56]. Other important transcription factor networks in PMNs include RUNX1 and KLF6, which modulate neutrophil maturation, RELB, IRF5, and JunB, which drive neutrophil effector responses; and RFX2 and RELB, which promote neutrophil survival [46]. These findings can potentially pave the way for the manipulation of distinct neutrophil subsets acting on perhaps disease- or tissue-specific transcriptional hubs. We refer readers to detailed reviews on neutrophil transcriptional heterogeneity for more information [47,53,67].

Aside from transcriptomic approaches, classifying neutrophils based on their separation on a Ficoll density gradient has revealed an important subpopulation called low-density granulocytes (LDGs) [68]. LDGs are a heterogenous population comprising different stages of proliferating immature and non-proliferative mature neutrophils in the bone marrow and are released as part of the stress granulopoiesis response. Ex vivo, LDGs, because they are enriched in developing neutrophils, display defective respiratory burst and NETosis [62]. However, their capacity for cytokine production is augmented, suggesting an immunoregulatory role for these cells [62]. However, because LDGs comprise such heterogenous neutrophil states, it is difficult to circumscribe them within a specific effector function or functions. For example, LDGs are pro-inflammatory and interferonogenic in SLE [69,70], whereas they suppress T cell responses in cancer [71], indicating a context-specific inducible gene expression signature. The regulation of how disease-specific LDG transcriptional signatures and functions are brought about and at which stage of the neutrophil compartment architecture these signals act (i.e., at the level of the bone marrow, circulation, or tissue) is investigational.

## 3. Neutrophils as Orchestrators of the Immune Response

**Figure 1:**
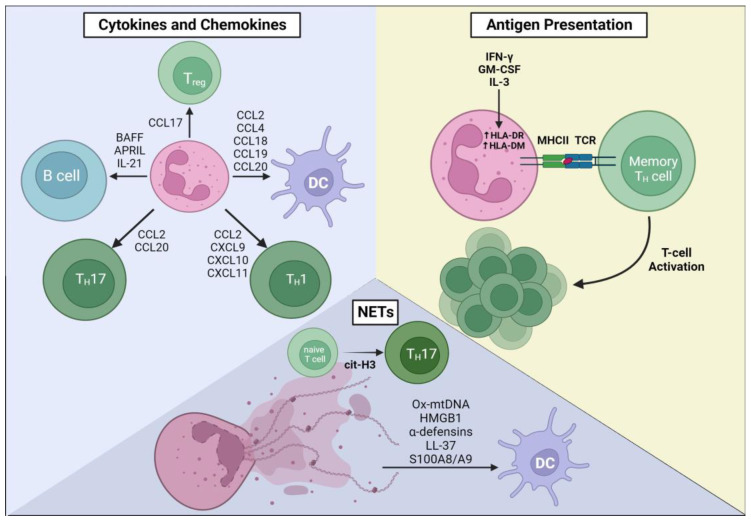
Neutrophil functions that are important for shaping the adaptive immune response to infectious or non-infectious challenges can be divided into three main functions, as are later discussed in this review. Firstly, neutrophils can present antigens to CD4^+^ T helper cells in the context of class II MHC. This appears to be relevant only to memory T helper cells. Neutrophils can release a myriad of cytokines and chemokines that recruit and modulate the activation of DCs and different subsets of T helper cells. Neutrophil-derived BAFF and APRIL enhance B-cell survival, maturation, and antibody production in a T cell-independent manner. Within NETs, alarmins can activate DCs, and histones can signal the TLR2-mediated differentiation of naïve CD4^+^ T cells from T_H_17 cells.

This section divides the immune-orchestrating functions of neutrophils into antigen presentation, the production of cytokines and chemokines, and the release of NETs. These functions are also summarized in Figure 1.

### 3.1. Antigen Presentation

Macrophages, DCs, and B cells are the three professional antigen-presenting cells (pAPCs) based on the expression of class II MHC on their surface on which they present antigens to CD4^+^ T cells [72]. APCs also express intracellular antigen-processing machinery that complexes antigens to class II MHC and costimulatory molecules like B7 (CD80/CD86) required for CD4^+^ T-cell activation [72]. By contrast, atypical APCs inducibly express these molecules in cells under stimulation by immunomodulatory factors like IFN IFNγ [73]. Almost all the granulocytes (neutrophils, basophils, mast cells, and eosinophils) and endothelial cells and many epithelial and stromal cell populations can upregulate class II MHC in certain settings, acting thereby as atypical APCs. For a detailed review of atypical APCs, see [73].

In homeostasis, neutrophils do not express class II MHC or costimulatory molecules CD80/CD86. Cytokines like GM-CSF, IFNγ, and IL-3 stimulate neutrophils to express HLA-DR (a gene encoding class II MHC) [74,75,76,77,78,79,80,81,82,83]. Incubating neutrophils with these cytokines results in HLA-DR upregulation and proliferation of antigen-specific effector T cells, indicating successful antigen presentation [84]. Polak et al. reported that cytokine-stimulated neutrophils upregulate HLA-DR and HLA-DM (involved in antigen processing) and process external antigens much faster than macrophages and DCs [85]. Cytokine-activated neutrophils also upregulate costimulatory molecules like CD80/CD86, which bind CD28; CD40, which binds CD40L; and CD58 (which is constitutively expressed), which binds CD2, all of which form integral components of the immunological synapse between pAPCs and CD4^+^ T cells to induce T cell activation and proliferation [84,86,87,88]. 

The short lifespan (<24 h) of neutrophils in blood appears to be incompatible with the length of time required for antigen processing, lymph node migration, antigen presentation, and activation of naïve T cells [89]. It presently seems that this process is restricted to memory T cells that require a short duration of T-cell receptor stimulation, suggesting its relevance in secondary immune responses; this seems counterintuitive given that neutrophils are first-line responders to infections, while macrophages and DCs, being professional APCs, drive the late response. The antigen-presenting function of neutrophils may be important for immune memory to vaccines, as indicated by neutrophils isolated from macaques immunized with the HIV-1 envelope glycoprotein that can present the vaccine antigen to memory T cells and induce their proliferation ex vivo [86]. Atypical APC-like neutrophils, when pulsed with the pollen allergen Bet v 1, induce proliferation and cytokine responses by allergen-specific T cells ex vivo, confirming that neutrophils successfully present antigens and that this could be relevant in late-phase allergic reactions [85]. An in vivo example of a neutrophil-mediated secondary adaptive immune response is the engraftment of human allergen-specific T cells into mice to induce airway hyperresponsiveness upon allergen exposure, which is significantly enhanced upon the co-injection of cytokine-activation HLA-DR^+^ neutrophils [87].

However, the lifespan of APC-like neutrophils may be extended in naïve tissues and in the presence of cytokines like TNF-α and GM-CSF, for instance, in the synovium of inflamed joints affected by rheumatoid arthritis or in the TME, which may allow antigen presentation to co-localizing naïve CD4^+^ T cells [8,44,81,90]. Neutrophils have previously been shown to migrate to lymph nodes following the capture of tumor antigens [91], but whether they successfully present these antigens to CD4^+^ T cells is yet to be proven. Cytokines like GM-CSF, IFN-γ, and IL-3 can be elevated in the TME; therefore, neutrophil reprogramming towards the atypical APC phenotype is plausible. In non-neoplastic contexts, HLA-DR^+^ CD80/86^+^ neutrophils are significantly more abundant in the blood of hyperlipidemic patients compared to non-hyperlipidemic controls [92]. Mechanistically, neutrophils activated by phorbol-12-myristate-13 acetate (PMA) in the presence of oxidized LDL—the initiator of atherosclerosis—acquire an APC-like phenotype with HLA-DR and CD80/86 upregulation. Furthermore, HLA-DR^+^ and CD80/86^+^ neutrophils are enriched in the atherosclerotic plaques of LDL receptor-deficient mice, and their presence is positively correlated with the local enrichment of IFNγ-producing CD4^+^ T cells. Co-culturing naïve T cells with these atypical APC neutrophils promoted T-cell proliferation and IFNγ production, which could be attenuated by HLA-DR/CD80/CD86-blocking neutralizing antibodies [92]. T_H_17 cells are also enriched in atheromas, where they produce cytokines/chemokines (IL-17, CXCL8, IFNγ, TNF, and GM-CSF) that promote the recruitment, activation, and survival of neutrophils [93,94,95]. Neutrophils stimulated in vitro by IFNγ plus lipopolysaccharide (LPS) can secrete CCL2 and CCL20, which are chemokines for T_H_17 cells (via binding to CCR2 and CCR6, respectively) [96]. These results suggest that T cells and neutrophils can set up a self-reinforcing positive feedback loop that amplifies neutrophil and T-cell recruitment and activation.

For future studies, it will be important to identify the cytokines (e.g., IFNγ) that synergize with oxLDL to promote APC marker upregulation in neutrophils, as well as the antigens presented by these neutrophils. Secondly, neutrophil functions like NETosis and ROS/degranulation may also contribute to atherosclerosis [97,98,99]. Discerning the relative importance of neutrophils as antigen-presenting cells against these other functions is important for the development of interventions targeting causal mechanisms rather than those that are simply associated. This highlights an important caveat when evaluating current data: although HLA-DR^+^ neutrophils can be seen in various disease contexts, overt evidence of their actionable value in terms of disease-modifying therapeutic strategies is still awaited.

### 3.2. Cytokine and Chemokine Production

The chemokines produced by neutrophils can recruit more neutrophils (CXCL1, CXCL2, CXCL5, and CXCL8), pAPCs like DCs (CCL2, CCL3, CCL4, CCL18, CCL19, and CCL20) and monocytes/macrophages (CCL2, CCL3, and CCL4), naïve T cells (CCL18), TH_1_ cells (CCL2, CXCL9, CXCL10, and CXCL11), T_H_17 cells (CCL2 and CCL20), and T_reg_ cells (CCL17) [100].

The specific chemokine pool contributed by neutrophils is stimulus dependent. For example, granulomatous infectious diseases like *Mycobacterium tuberculosis* and *Schistosoma japonicum* feature a neutrophil chemokine signature that recruits DCs, monocytes, and CD4^+^ T_H_1 cells to promote granuloma formation [101,102,103]. Neutrophils produce CXCL10, CCL2, and CCL20 to recruit T_H_1 and T_H_17 cells upon Borrelia burgdorferi infection [104]. In viral infections, neutrophils incubated with respiratory syncytial virus secrete CCL3, CCL4, and CXCL8, indicative of their pro-inflammatory role in bronchiolitis caused by this infection [105,106,107,108]. In herpes simplex virus-1 (HSV-1)-induced keratitis (i.e., corneal inflammation), neutrophils elicit CXCL10-dependent T_H_1 responses that, albeit while suppressing viral replication and HSV-1 titers, do not limit the spread of infection to contiguous vital structures like the retina [109]. Other infectious agents against which cell-mediated immunity is crucial have evolved mechanisms of reducing the production of T_H_1-favoring chemokines CXCL9 and CXCL10 by neutrophils [110,111].

Regarding cytokines, neutrophils synthesize and release various pro-inflammatory, anti-inflammatory, and immunomodulatory (IFNβ, IFNγ, IL-12, IL-21, IL-22, and IL-23) cytokines (Table 1) [2,100]. Although rodent neutrophils secrete IL-17, human neutrophils do not appear to do so [2,112]. As with chemokines, context is essential in determining the neutrophil contribution to the cytokine milieu. For instance, in *M. tuberculosis* infection, neutrophils localize to multiple spatially distinct compartments of tuberculous granulomas, where they secrete both pro-inflammatory (TNF-α and IFNγ) and anti-inflammatory cytokines (IL-4 and IL-10) [113]. In COVID-19, neutrophils contribute to disease pathogenesis by producing pro-inflammatory cytokines like TNF-α and IL-6 that contribute to lung-centric and systemic cytokine storms and the chemokine CXCL8 to promote further neutrophil recruitment and augment inflammation [114,115,116,117,118,119]. 

Neutrophils can also produce cytokine members of the TNF superfamily, like B-cell activating factor (BAFF) and a proliferation-inducing ligand (APRIL) to accelerate B-cell maturation. Indeed, neutrophils colonizing the marginal zone of the spleen release cytokines like BAFF, APRIL, and IL-21 to promote B-cell maturation into plasma cells, somatic hypermutation, and antibody secretion [58]. Neutrophil-derived BAFF is a pathway for T cell-independent antibody production upon West Nile Virus infection [130]. Similarly, neutrophils recruited to lymph nodes via IL-17 during chronic infections can produce BAFF to accelerate plasma cell generation and antigen-specific antibody production [131]. In the intestinal mucosa, signaling through receptors for the anaphylatoxin complement proteins C3a and C5a induces BAFF production by neutrophils for T cell-independent B-cell expansion and antibody production [132]. This is crucial to the formation of B-cell germinal centers within Peyer’s patches and IgA class switching. In the context of vaccination, simian immunodeficiency virus (SIV) vaccination in rhesus macaques enhances the capacity of neutrophils for B-cell help, with the isolation of B-cells with neutrophils from vaccinated animals increasing B-cell differentiation, antibody production, and class switching [133].

These cytokines and chemokines are in addition to the immunomodulatory products that neutrophils produce. Neutrophil products like lactoferrin and α-defensins are alarmins and chemotactic for DCs and are essential for their recruitment to sites of infection [134]. Neutrophil-derived ROS can either stimulate or suppress T-cell responses [135]. Arginase-1 is a neutrophil-derived granule protein that depletes extracellular arginine to potently inhibit T-cell activation [136,137].

### 3.3. Neutrophil Extracellular Trap Production

This section briefly highlights the impact of NETs on pAPCs and adaptive immune cells. In addition to this, NETs play key prothrombotic and antimicrobial roles that have been discussed in detail in other reviews [6,7,138].

NETs can directly link innate and adaptive immune responses by physically contacting and reducing the activation threshold of T cells, enabling their activation to even subimmunogenic stimuli [139]. In addition, histones within NETs can signal the TLR2-dependent phosphorylation of STAT3 and activation of the transcription factor RORγT, which is key to the differentiation of naïve T cells into T_H_17 effectors [140].

NETs are internalized and thereby cleared by macrophages and DCs cells [141]. The internalization of NETs by macrophages and DCs into phagosomes activates the cytosolic DNA sensor cyclic GMP-AMP synthase (cGAS) and type I IFN production, which plays a role in antiviral immune responses and autoimmunity [142]. Conversely, NETs can attenuate the LPS-induced activation of DCs and decrease their expression of HLA-DR and CD80/86, thus reducing their ability to activate CD4^+^ T cells [143]. Neutrophil-derived MPO can directly reduce DC-mediated T-cell activation, with its depletion increasing T cell-mediated allergic inflammation and antigen-induced arthritis [144].

Alarmins, molecules released by cells upon cell death, are a subset of damage-associated molecular patterns (DAMPs) that can activate pattern recognition receptors (PRRs) in immune cells [145]. Most alarmins studied to date induce the recruitment and activation of antigen-presenting cells like macrophages and DCs, rendering them a critical link between innate and adaptive immunity [145,146]. The alarmin S100A8/A9 (also known as calprotectin) constitutes about 40% of neutrophilic cytosolic content and decorates NETs [147,148]. Other classical alarmins contained within NETs include DNA, DNA-associated proteins like high-mobility group box-1 (HMGB1), α-defensins, and LL-37/cathelicidin [149,150]. The release of these alarmins within the highly concentrated, antigen-sequestrated environment of NETs is likely to recruit and activate DCs, including plasmacytoid DCs (pDCs) that produce type I IFNs [134,151]. Additionally, LL-37 released by neutrophils via degranulation, NETosis, or necrotic death can direct CD4^+^ T cells towards a T_H_17 phenotype in the presence of TGF-β [152]. Alarmins like HMGB-1 and cathelicidin also can exert autocrine effects on neutrophils to promote further NET formation [153,154].

## 4. Pro-Homeostatic Roles of Neutrophils

The bone marrow commits most of its anabolic capacity into granulopoiesis [155], which is counterintuitive given the short lifespan of these cells, their unclear role in homeostasis, and the very low likelihood that most of these cells will encounter a microbe during their life. Recent data from multi-omic approaches and Ly6G reporter mice show that, during homeostasis conditions, circulating neutrophils home into most naïve tissues and adopt a tissue-resident phenotype, with a similar or even longer lifespan than their circulating counterparts [44,48]. How tissue-resident neutrophils contribute to homeostasis, for instance, by complementing the role of tissue-resident macrophages, is not known. 

A novel role for neutrophils as mediators of communication between organs is now emerging in tandem with data that now show neutrophils to be capable of moving out of tissues and re-entering circulation [156]. This may be a double-edged sword in diseases, as one the one hand it would promote resolution by clearing neutrophils from the affected site but it could lead to systemic spread of the inflammatory response on the other [157]. By way of example, during conditions of homeostasis, aged neutrophils at the end of their lifespan are phagocytosed by macrophages in the bone marrow, which, in turn suppresses the release of hematopoietic stem cells to establish a rhythmic/circadian release of hematopoietic progenitors into circulation [158]. In contrast, in the context of myocardial infarction, IL-1β enriched in the ischemic myocardium can activate the NLP3 inflammasome to prime neutrophils, which then reverse migrate into circulation and the bone marrow to release IL-1β and stimulate granulopoiesis [159].

In disease states, the pro-homeostatic role of neutrophils is exemplified by their complex role in wound healing. In general, tissue injury—be it traumatic, ischemic, or infectious—is sensed by the immune system, which then seeks to eliminate the inciting cause and re-establish homeostasis. Being the first cells recruited to wound sites, neutrophils stand at the frontline of this response as they phagocytose and clear inflammatory debris to decontaminate the wound site [160]. This paves the way for the subsequent proliferation and remodeling phases of wound healing. In skin wounds, neutrophil effector functions like phagocytosis, ROS production, degranulation, and NETosis additionally prevent microbial colonization that would otherwise precipitate a non-healing wound [161]. After enacting their phagocytic and microbicidal effector functions, neutrophils become apoptotic and are engulfed by macrophages by efferocytosis, which initiates a switch in macrophage polarization from pro-inflammatory to anti-inflammatory/pro-repair [162]. In agreement with these findings, abrogating neutrophil recruitment to wound sites in genetically modified CXCR2-knockout mice delays the healing of cutaneous wounds [163]. A beneficial role of neutrophils in wound healing is also supported by the fact that aging-associated impairment in wound healing has been related to aged neutrophils being less effective in phagocytosis and respiratory burst and to impaired macrophage efferocytosis of neutrophils, resulting in a prolonged inflammatory phase [164,165,166,167].

Neutrophils play a key role in orchestrating cardiac healing after myocardial infarction, separate from their widely recognized role as detrimental mediators of ischemia/reperfusion injury [168]. Transcriptomic and proteomic studies indicate that neutrophils switch from a pro-inflammatory to anti-inflammatory/pro-repair as the inflammation phase of wound healing resolves and the proliferative/remodeling phases begin [66,169]. The first wave of neutrophils infiltrating the myocardium in the acute phase post-infarction encounter endogenous DAMPs that activate surface TLR4 and induce pro-inflammatory (formerly N1) states expressing high levels of IL-1β, IL-6, TNF-α, and matrix metalloproteinases (MMPs). The magnitude of this response is directly associated with the thinning of the left ventricular (LV) wall and consequent dilatation of the LV cavity. However, the abundance of anti-inflammatory (formerly N2) neutrophils expressing IL-10 and CD206 progressively rise during the subacute phase of MI [66]. An important feature of transcriptomic reprogramming of neutrophils by the microenvironment of the ischemic myocardium is the upregulation of SiglecF that increases during the subacute phase of MI [170], which may be associated with neutrophil apoptosis and the resolution of inflammation. Neutrophils may also polarize macrophages to an anti-inflammatory/pro-repair phenotype and allow them to become more efficient efferocytes in the setting of myocardial infarctions in mice by secreting gelatinase-derived lipocalin (NGAL) [171]. In support of this, neutrophil-depleted mice have increased microphage proliferation at the myocardial infarct site but lower expression of MERTK in macrophages (a phagocytosis receptor) with decreased clearance of apoptotic cardiomyocytes, phenotypically manifesting as worse cardiac function post-infarct with increased fibrosis, whereas MERTK expression could be restored in vitro in the presence of NGAL [171].

A key function of neutrophils in supporting wound healing and tissue restoration is angiogenesis. During steady state, circulating neutrophils destined for angiogenic functions express surface CXCR4, which responds to a CXCL12 chemoattractant gradient that recruits these cells to the lungs to participate in vascular repair [44]. The CXCL12/CXCR4 signaling axis is also implicated in retaining angiogenic neutrophils at sites of inflammation, like chronic lung disease or inflammatory bowel disease [172,173]. Alternatively, the upregulation of VEGF-A at a site of ischemic injury can recruit neutrophils in a manner dependent on the neutrophil integrin VLA-4 [174,175]. Other than direct VEGF secretion, neutrophil secretion of MMP-9 to liberate matrix-bound VEGF is another important mechanism by which these cells contribute to angiogenesis [176,177,178]. Whether there is a role for angiogenic neutrophils in naïve tissues (i.e., in the absence of injury) outside of the lungs remains to be seen. Additionally, therapeutically augmenting CXCR4^+^/VEGFR1^+^ neutrophils to improve revascularization and tissue restoration in cases of ischemic injury or organ transplantation and, by the same token, mitigating angiogenic neutrophils to counteract their potential pro-tumorigenic role without interfering with the physiological role of these cells in the lungs are outstanding goals for future research.

It should be kept in mind that these tissue-restorative functions of neutrophils are regulated by factors like age and comorbidities like diabetes, which are known to perturb neutrophil effector functions to promote chronic wound pathology [179]. Similarly, NETs constitute a double-edged sword in inflammation, being shown to trap microbial pathogens and enhance their killing on the one hand and exacerbating inflammation in COVID-19, impaired wound healing, tumor progression, and autoimmunity on the other [5,138,180,181,182,183]. Regarding their beneficial role, NETs may clear diseased/senescent endothelial cells in diabetic retinopathy [184]. Sites densely infiltrated by neutrophils can feature the production of aggregated NETs (aggNETs). The proteases within aggNETs are entrapped within the NET matrix, which not only shields them from normal adjacent tissues but also protects them against antiproteases, thereby extending their lifespan. However, cytokines and chemokines are still accessible to NET proteases and can be degraded to allow inflammation resolution [185]. This is evident in gouty arthritis, where aggNETs induced by monosodium urate (MSU) crystals can degrade inflammatory mediators [186]. AggNETs can also degrade extracellular histones, preventing their toxic effects on epithelial and endothelial cells that otherwise would lead to microvascular dysfunction and thrombosis [187].

## 5. Role of Neutrophils in Immune-Related Diseases

### 5.1. COVID-19

A central mechanism by which SARS-CoV-2 causes severe disease is by delaying the production of type I IFNs, which are key initiators of antiviral immunity [188,189,190]. This delay in type I IFN responses and T-cell recruitment is associated with an exaggerated myeloid cell—particularly neutrophil—response [191,192,193]. Salient features of severe COVID-19 include an elevated neutrophil-to-lymphocyte ratio [194], the emergence of immature neutrophil populations in both the blood and lungs [195,196,197], and activated pro-inflammatory neutrophil phenotypes in both the circulation and lungs with an enhanced production of TNF-α, IL-6, CXCL8, alarmins like calprotectin (SA100A8/A9), and NETs [198,199,200]. Through these mechanisms, neutrophils contribute to local tissue damage, amplify lung inflammation, and activate platelets and the coagulation cascade via NETs to promote thrombosis [201,202,203,204].

Profound metabolic derangements are observed in neutrophils isolated from severe COVID-19 patients who develop acute respiratory distress syndrome (ARDS) and require intensive care unit (ICU) admission [205]. These include a depletion of intracellular histidine, higher β-alanine, and elevated oxidative stress, but the impact of these changes on neutrophil function is not known. In addition, the activity of the glycolytic enzyme glyceraldehyde-3-phosphate dehydrogenase (GAPDH) is reduced in patients with severe COVID-19, which decreases glycolysis and increases the activity of the HMP shunt. Functionally, the decrease in GAPDH activity is linked to increased NET production, but this is independent of its effects on increasing HMP shunt activity and NADPH production [205].

The transcriptomic landscape of circulating neutrophils is significantly dysregulated in severe COVID-19, with the emergence of immature and dysfunctional neutrophils and PD-L1^+^ neutrophils and loss of IFN-active neutrophils [199,206,207]. PD-L1^+^ neutrophils decrease in patients recovering from severe disease, whereas IFN-active neutrophils appear in both mild and severe COVID-19 [208,209]; hence, their relevance to the disease process is currently unclear. These findings likely mirror the temporal dynamics of type I IFN responses in varying disease severities. A failure to develop early type I IFN responses is associated with severe COVID-19, but conflicting evidence suggested that type I IFNs and their stimulated genes are elevated in severe COVID and correlate directly with mortality and levels of pro-inflammatory cytokines like TNFα and IL-6 [188,209,210,211,212]. Many of these differences could be partly explained by between-study variations in definitions of severe disease, times of sampling, and methods to measure type I IFNs. A recent study more definitively showed that levels of type I IFNs are indeed decreased in severe COVID-19 [213]. A model of COVID-19 pathogenesis from an IFN perspective states that their production in early-stage disease results in timely antiviral responses and the recruitment of adaptive immunity, whereas their delayed production in severe COVID-19, as a consequence of a profoundly dysregulated immune system, may enhance innate immune inflammation [214]. This was demonstrated by an ex vivo stimulation of the whole blood of COVID-19 patients by type I IFN, which induced an inflammatory response in leukocytes of severe COVID-19 patients but not in those with mild/moderate disease [213]. Dexamethasone treatment to alleviate immunopathology of severe COVID-19 has been shown to induce the depletion of IFN-stimulated neutrophils [208], while the high level of NET production by neutrophils escapes modulation by dexamethasone treatment [215]. These findings indicate that combining conventional immunosuppression with NET-targeting therapies may enact a synergistic effect on attenuating COVID-19 hyperinflammation and immunopathology.

Long COVID, also known as post-acute COVID-19 sequelae (PACS), describes the multi-system, non-specific symptoms that survivors of COVID-19 develop after the resolution of the acute phase infection. It has thus far proven difficult to identify universally conserved pathophysiological hallmarks of PACS due to significant biological and clinical disease heterogeneity. Chronic immune dysregulation affecting the innate and adaptive response is a salient yet contentious feature of PACS and may be brought about by SARS-CoV-2 viral persistence/viral reservoirs, reactivation of latent viruses like varicella zoster virus (VZV) and/or Epstein–Barr virus (EBV), autoimmunity, and gut microbiome dysbiosis [216,217]. Neutrophil-derived calprotectin, a marker of neutrophil activation and degranulation, and citrullinated histone H3 (cit-H3), a marker of NET production, are enriched in a subset of patients with so-called ‘inflammatory PACS’ [218]. In the postmortem lung specimens of patients who died from COVID-19, immature neutrophils were seen co-localizing with CD8^+^ T cells and regenerating alveolar epithelium in areas of diffuse alveolar damage [197], suggesting that perhaps neutrophils could be playing a role in the persistent stimulation of T cells or impairing the regeneration of alveoli that could be relevant to the chronic pulmonary sequelae of fibrosis that survivors develop [219]. 

Autoantibodies are known to emerge in severe acute COVID-19 and also in PACS, although their role in the chronic sequela was cast into doubt by a study applying multidimensional immunophenotyping and unbiased machine learning approaches to study conserved immune trajectories in 275 PACS patients [220]. Nevertheless, keeping in mind the biological heterogeneity of PACS, severe COVID-19 patients can develop IgG and IgM antibodies against NETs, which can protect these structures against degradation by circulating DNase [221]; their persistence in survivors of severe COVID-19 who develop PACS may contribute to inflammatory PACS [222]. A fraction of PACS patients and approximately half of hospitalized COVID-19 patients can develop autoantibodies typical of antiphospholipid syndrome, such as anti-cardiolipin, anti-phosphatidylserine, and anti-β_2_ glycoprotein [223]. These antibodies trigger NETosis when injected into mice [223], and PACS patients exhibiting positivity for these antibodies demonstrate higher levels of NET markers than healthy controls [224]. Phenotypically, the persistence of NETs in PACS may contribute to chronic lung injury and aberrant regeneration with fibrosis [225]. Together, these findings indicate that neutrophil effector functions like NET production can be present in a subset of PACS patients [226], but further studies are required to substantiate these observations.

### 5.2. Cancer

Complement proteins, cytokines, and chemokines like C3a, C5a, GM-CSF, CXCR1/2 agonists, IL-8, IL-17, and IL-1β within the TME (the specific cytokine/chemokine composition varies between tumors) can recruit neutrophils and reprogram them towards pro-tumor (N2 or PMN-derived suppressor cells (PMN-MDSCs)) or antitumor (N1) functionalities [227,228]. N1 TANs display greater cytotoxicity against tumor cells and activate antitumor T cells and natural killer cells, whereas N2 and PMN-MDSCs favor M2 macrophage polarization and T_reg_ differentiation to suppress antitumor immunity [229]. It should be mentioned that it is unclear whether N2 TANs and PMN-MDSCs represent distinct neutrophil populations. A recent consensus statement suggested dropping the terminology of PMN-MDSCs and instead considering them both under protumor TANs [230]. Mounting evidence of neutrophil plasticity/heterogeneity also supports moving past these terminologies.

The notion that TANs can adopt heterogenous phenotypes has been extensively studied in hepatocellular carcinoma (HCC) [231]. Indeed, 12 transcriptionally distinct TAN subtypes have been described in human HCC [232]. Functionally, PD-L1^+^ TANs in the HCC TME can suppress antitumor CD8^+^ T-cell responses [233,234]. In addition, HCC-associated TANs can elaborate CCL2, CCL3, and CCL17 to recruit immunosuppressive macrophages and T_regs_ [232,235]. In contrast, in lung cancer models, TANs can orchestrate potent antitumor immune responses. In this regard, TANs can upregulate class II MHC to stimulate antitumor T-cell responses that can mitigate the tumor progression of early-stage lung cancer [236,237]. The composite of cytokines/chemokines released by N1-like TANs like CCL3, CCL9, TNF-α, and IL-12 can recruit and activate T_H_1 and CD8^+^ cytotoxic T cells to exert antitumor effects [238]. TAN-derived ROS can directly inhibit IL-17 release by pro-tumorigenic γδ T cells [239].

Clinically, the anti-angiogenic treatment sorafenib—one of the first-line agents to treat HCC—is associated with an increase in TANs that produce CCL2 and CCL17, which can establish immunosuppressive niches within the TME to promote tumor cell survival. Accordingly, inhibiting transcriptional regulators of CCL2/CCL17, like p38 and AKT, significantly improved tumor sensitivity to sorafenib [235]. Along similar lines, cabozantinib (anti-angiogenic) and anti-PD1 immune checkpoint inhibitor combination therapy showed increased antitumor efficacy compared to monotherapy with either agent [240]. The combination regimen worked synergistically to increase circulating T-cell numbers, decrease the neutrophil-to-lymphocyte ratio, and decrease intratumoral exhausted PD1^+^ CD8^+^ T cells and T_regs_. Interestingly, human HCCs that exhibited a favorable response to treatment showed an increased TAN presence in the TME [240], suggesting that anti-angiogenic and immune checkpoint therapies can reprogram TAN-driven innate and adaptive responses in the TME towards antitumor immunity. Indeed, anticancer immunotherapies can increase a specific subset of IFN-active TANs that were associated with enhanced antitumor immunity [241].

Regarding the humoral response, B cells recruited to the TME by TAN-derived TNF-α can become activated and differentiate into IgG-producing plasma cells independent of T-cell interactions [242]. This was shown to be predicated on cell-to-cell contact with TANs and driven partly by TAN-derived BAFF [242]. The bone marrow environment in multiple myeloma is characterized by inflammasome-primed neutrophils that secrete BAFF to enhance myeloma cell survival and maintain a pro-inflammatory marrow microenvironment [243,244]. This environment can persist after the treatment of multiple myeloma with hematopoietic stem cell transplant, suggesting its role in disease recurrence.

NET release is increasingly being shown to be playing a key role in the TME. The chromatin-based matrix of NETs can act as a physical barrier that prevents contact between tumor cells and antitumor CD8^+^ T cells and natural killer cells [245,246,247]. Furthermore, PD-L1 embedded within NETs can induce exhausted phenotypes in CD8^+^ T cells in murine models of liver metastasis [248]. In a clinical context, a risk score composed of six NETs-related genes could estimate the prognosis of colon cancer patients and their response to immunotherapy [249]. In addition, NET-derived MPO spatially associates with T_regs_ and PD-1^+^ T_regs_ that attenuate responses to immune checkpoint therapies [249]. PAD4 inhibition to attenuate NET formation increases the sensitivity of pancreatic cancer to immune checkpoint inhibitors [246]. NETs can modulate tumor responsiveness to the more conventional lines of cancer treatment too. Radiation treatment of bladder cancer increases the production of NETs in the TME, which prevents antitumor CD8^+^ cytotoxic T cells from having access to residual tumor cells, an effect that could be alleviated by degrading NETs via DNase-1 [250]. Chemotherapy-treated gastric cancer cells elaborate IL-1β to stimulate NET production that confers chemoresistance by increasing local TGF-β concentration [251]. Moreover, NETs-mediated immunosuppression may be one of the direct facilitators of HCC development by TLR4-mediated metabolic reprogramming of naïve T cells to T_regs_ [252]. Blocking NETs in vivo using PAD4-knockout mice or DNAse-1 reduces the numbers of T_regs_, significantly attenuating the progression of non-alcoholic steatohepatitis into HCC [252].

Multiple lines of evidence show that NETs can augment antitumor immunity. For example, intravesical BCG therapy for bladder cancer recruits TANs and stimulates NET release, which, in turn, enhances the recruitment of antitumor monocytes and T cells to the TME [253]. In melanoma, NETs adhere to tumor cells via integrins to prevent their migration and exert direct cytotoxicity [254]. Melanoma-specific T-cell immunotherapies and immune checkpoint inhibitors significantly increase antitumor immunity against melanoma cells in the early phase of treatment. However, complete tumor eradication, including the killing of antigen loss variants, during late-phase treatment is dependent on neutrophil recruitment and NET production [255].

These phenotypes of TANs are partially due to metabolic reprogramming. The depletion of extracellular glucose in the TME, as well as factors derived from tumor cells like GM-CSF in select tumor models, can cause TANs to upregulate fatty acid uptake, reprogramming them towards a phenotype of protumor TANs that suppress antigen-specific antitumor CD8^+^ T cells [256,257,258,259,260,261]. The TME is also highly heterogeneous in terms of its blood supply, oxygen and glucose availability, cytokine/chemokine/growth factor milieu, and immune and stromal cell composition and phenotype. Further layers of complexity can be added when considering the specific organ affected. It is likely that neutrophils in spatially distinct compartments of the TME are driven to diverging phenotypes that co-exist and partake in homotypic and heterotypic cellular interactions to shape the immune response to tumors, either towards antitumor immunity or immunosuppression. A spatial understanding of neutrophil identity in the TME combined with single-cell approaches would provide more insights into understanding their divergent individual phenotypes in the TME.

Another aspect of tumor biology that influences tumor response to immunotherapy is the genomic makeup of tumors. Performing immunogenomic analyses of 67 spatially distinct regions of an anti-PD-1-resistant melanoma sample, Mitra et al. reported that tumor regions harboring a subclonal gain of chromosome 7 exhibit an accumulation of immunosuppressive TANs in the TME, the transcriptomic signature of which was associated with resistance to immune checkpoint inhibitors like anti-CTLA4 and anti-PD-1 [262]. Therefore, both tumor-extrinsic factors, like blood flow and nutrient and oxygen availability; and intrinsic factors like genomic abnormalities may influence the abundance and activation phenotypes of TANs.

### 5.3. Autoimmune Diseases

Autoimmune diseases are characterized by a loss of tolerance, leading to the failure of the adaptive immune system to distinguish self from non-self-antigens and consequently causing it to attack host tissues. Neutrophils in the blood of patients with systemic autoimmune diseases exhibit an activated phenotype and are present at inflamed sites like the vascular wall in vasculitis, the kidney in SLE, and the synovium in rheumatoid arthritis [263]. At these sites, neutrophils enact their classical effector functions of phagocytosis, degranulation, and NET release, as well as their immunoregulatory roles of cytokine/chemokine production and antigen-presentation. NETs constitute a rich source of intracellular alarmins, like HMGB1, that are normally shielded from the immune system. The release of these alarmins results in antigen sequestration and increased autoimmune activity [134]. Additionally, immune complexes (ICs), the presence of which is a cornerstone of many autoimmune diseases, can stimulate the production of lytic NETs or non-lytic mitochondrial DNA-containing NETs [70,264]. Lastly, autoimmune diseases like SLE are associated with an increase in T_H_17 responses [265], and this may be related to NET production as cit-H3 within NETs can directly stimulate T_H_17 differentiation via TLR2 signaling [140].

Neutrophils are abundant in the synovial fluid of inflamed joints in rheumatoid arthritis, juvenile idiopathic arthritis, and monosodium urate crystal-induced arthritis [266,267,268]. Functionally, chemokines upregulated by synovium-infiltrating neutrophils are associated with the chemoattraction of more neutrophils (CXCL1, CXCL2, and CXCL8), DCs (CCL2, CCL4, and CXCL16), T_H_1 cells (CCL2 and CXCL10), and T_H_17 cells (CCL2 and CCL20) [96,269]. Synovial-fluid neutrophils in rheumatoid arthritis express and secrete BAFF and APRIL that could promote autoantibody production [270,271]. Rheumatoid arthritis patients typically exhibit positivity for anti-citrullinated peptide antibodies (ACPAs), and this positivity correlates with disease progression [272]. Inoculating control neutrophils with synovial fluid from rheumatoid arthritis patients elicits NET production with exposure to cit-H3-positive DNA [269], indicating that NETs can constitute a major source of citrullinated peptides against which antibodies are specifically generated in rheumatoid arthritis [273,274]. In support of this, serum from RA patients who are positive for ACPA cross-reacts with the cit-H4 present within NETs [275]. Mechanistically, citrullinated proteins within NETs can be endocytosed by MHC II-expressing synoviocytes and presented to antigen-specific CD4^+^ T cells to initiate T cell-dependent B-cell maturation and autoantibody production [276]. Alternatively, NET-derived neutrophil elastase can damage the intra-articular cartilage of affected joints, liberating its proteins for citrullination and subsequent presentation by synoviocytes to CD4^+^ T cells [277]. Considering these findings with the observations that (1) neutrophils are one of the most abundant cells in the synovium of joints affected by rheumatoid arthritis, (2) they can directly present antigens to CD4^+^ T cells, and (3) they stimulate B-cell differentiation and antibody production through BAFF/APRIL implicates neutrophils as being key to the loss of immune tolerance and production of pathogenic ACPA.

Neutrophils in SLE appear to be skewed towards LDGs, which are primed population with augmented pro-inflammatory activity and NETosis [278,279,280]. Ribonucleoprotein (RNP)-antibody ICs in SLE induce the secretion of BAFF by neutrophils that promote B-cell survival, proliferation, and plasmablast differentiation, indicating that perhaps neutrophils may sustain autoantibody production in lupus [281]. Neutrophils isolated from SLE patients display reduced NADPH oxidase-dependent ROS production [282]. These findings indicate an impairment of respiratory burst-dependent processes in SLE that play a key role in the phagocytic clearance of apoptotic cellular debris, thereby leading to the persistence of intracellular debris that can act as neoantigens or substrates for autoantibodies to form ICs [283]. Indeed, NADPH oxidase-knockout mice have a higher risk of developing SLE and progression of existing disease [284]. Similarly, pristane-induced lupus (PIL) was exacerbated in mice deficient in NADPH oxidase or PAD4, whereas treating PIL mice with NADPH oxidase activators induces lytic NET formation and ameliorated disease severity [285].

In contrast, LDGs stimulated by ribonucleoprotein (RNP)-ICs in SLE upregulate mitochondrial ROS production and the oxidation of mitochondrial DNA that is then extruded in oxidized mitochondrial DNA-containing NETs in an NADPH oxidase-independent manner [286,287]. The mechanism of NET formation in SLE was recently clarified. Circulating ICs activate the Fcγ receptor (FcγR) on the surface of neutrophils, leading to the inactivation of serpinb1 and consequent activation of intracellular caspase-1/caspase-11 that cleave and thereby activate gasdermin D [23]. Importantly, gasdermin D can mediate both lytic NETosis (with nuclear DNA) and non-lytic mitochondrial NET formation (decorated with oxidized mitochondrial DNA), whereas PAD4 is only involved in NADPH oxidase-dependent lytic NETosis (which may be defective in SLE—see above). Accordingly, the RNP-IC-mediated pathway of NET production induced mitochondrial ROS production and required gasdermin-D activation but not PAD4. This was confirmed pharmacologically in vitro, where PAD4 inhibitors (GSK484) had no effect on the release of RNP-IC-related NET release from control human neutrophils and LDGs derived from lupus patients, whereas gasdermin-D inhibition (disulfiram) or mitochondrial ROS attenuation (Mito-TEMPO) significantly attenuated oxidized mitochondrial DNA release within NETs [23].

Downstream, NETs containing oxidized mitochondrial DNA can trigger the TLR9-dependent activation of pDC, leading to the production type I IFNs that stimulate autoreactive T cells and lower the activation threshold of autoreactive B-cells [288]. NETs decorated with IL-33 also directly activate TLR7/9 signaling in pDCs and the production of type I IFNs [289]. Alternatively, the uptake of the oxidized mitochondrial DNA of NETs (promoted by NET proteins LL-37 and HMGB1) by pDCs activates the cGAS/STING pathway of type I IFN production in SLE [287,290]. Independently, LL-37-DNA complexes within NETs can directly get endocytosed by polyclonal B cells, activating TLR9 signaling, clonal expansion, and anti-LL-37 antibody production, which are autoantibodies seen in SLE patients [280]. The degradation of NETs can be impaired in SLE, as NET-directed autoantibodies like anti-dsDNA antibodies and circulating factors like C1q protect NETs against degradation in SLE, and genetic polymorphisms that negatively affect the function of DNase enzymes can increase the risk of the development of lupus [291,292,293,294,295,296]. This persistence of NETs may promote exposure to autoantigens and augment autoimmunity.

Together, these findings indicate that SLE immunopathogenesis may be initiated or exacerbated by an increase in the circulating proportions of LDGs prone to mitochondrial DNA-enriched NETs, which stimulate IFN responses in pDCs, as well as activate autoreactive B cells. What genetic, epigenetic, bone marrow, circulating, lifestyle, and/or environmental factors skew neutrophils towards LDGs to kickstart this whole process remain to be elucidated. Importantly, the link between mitochondrial DNA-containing NETs and pDCs with regards to type I IFN production may need to be revisited given recent data describing that pDCs may not be the major producers of type I IFNs in SLE, as evidenced by their defective production of type I IFNs in SLE [297]. Instead, nonhematopoietic cells like keratinocytes in cutaneous lupus and tubular epithelial cells of the kidney in lupus nephritis can be major producers of type I IFNs [297,298,299]. Furthermore, neutrophils can produce type I IFNs like IFNα in response to extracellular chromatin by upregulating cytosolic DNA sensors like the cGAS/STING pathway, which is associated with further NET production [300]. This suggests the presence of a positive feedback loop where NET production drives IFN production by neutrophils, thereby driving further autoimmune inflammation and NET production.

## 6. Conclusions

This review highlights the multifaceted role played by neutrophils, the first-line innate immune effector cells, in fine-tuning innate and adaptive immune responses. Better understanding these fine-tuning, or orchestrating, features of neutrophils is predicated on recognizing that transcriptionally and functionally distinct subpopulations of neutrophils likely coexist—their balance determined by the disease and tissue context—and work together during infectious and sterile states disease states. Understanding the spatial features of tissue-level neutrophil heterogeneity in both homeostasis and disease may enable a better comprehension of the upstream microenvironmental regulators of neutrophil heterogeneity and their downstream cellular interactions. It is plausible that, like macrophages, an imbalance between different neutrophil populations towards an end of a phenotypic spectrum, i.e., either in favor of inflammation or of tolerance, favors disease development or impairs disease resolution. This can either be manifested as exaggerated immune responses, as happens in autoimmunity or chronic inflammation, or a subdued adaptive response that may predispose to infections or tumorigenesis. Early evidence has indicated that there are candidate/core genes and transcriptional regulators that run a common thread behind either pro-inflammatory or immunomodulatory neutrophil populations. Whether or not these can be pharmacologically modified to alter disease pathogenesis is crucial to increasing the translational value of such research.

## Figures and Tables

**Table 1:** Pro- and anti-inflammatory cytokines produced by neutrophils.

Inflammatory	Pro-Homeostatic/Anti-Inflammatory
IL-1α [120]	IL-10 [113]
IL-1β [120]	IL-4 [121]
IFNγ [113,122]	IL-1RA [123]
TNF [124]	IL-22 [125]
IL-12 [126]	TGFβ [127]
IL-6 [128]	
IL-17 [129]	
